# Nurse–Physician Collaboration Scale: development and psychometric testing

**DOI:** 10.1111/j.1365-2648.2009.05011.x

**Published:** 2009-07

**Authors:** Rei Ushiro

**Affiliations:** School of Nursing, Jichi Medical UniversityTochigi, Japan

**Keywords:** instrument development, Japan, Nurse–Physician Collaboration Scale, psychometric testing

## Abstract

**Title:**

**Nurse-Physician Collaboration Scale: development and psychometric testing.**

**Aim:**

This paper is a report of a study conducted to develop and test the psychometric properties of the Nurse–Physician Collaboration Scale.

**Background:**

The importance of cooperation between healthcare professionals is widely acknowledged in Europe and the United States of America, but there have been no specific studies of interactions between healthcare professionals or of nurse–physician cooperation in Japan.

**Methods:**

The 51-item Nurse–Physician Collaboration Scale was developed using a process of item design, item refinement, and testing for reliability and validity. Random sampling was used to identify potential respondents from 27 of the 87 acute care hospitals in one city in Japan in 2006. Valid responses were obtained from 446 physicians and 1217 nurses (response rate 78·7% for nurses, and 54·4% for physicians). Construct validity was first confirmed by an exploratory factor analysis and then by a confirmatory factor analysis. Finally, a simultaneous analysis of several groups was performed. The test–retest method and Cronbach’s α coefficients were used to assess reliability.

**Findings:**

Exploratory factor analysis yielded three factors. The three-factor models were confirmed by a confirmatory factor analysis (CFI >0·9, RMSEA <0·08). Simultaneous analysis of several groups (RMSEA = 0·046, AIC = 3115·888) showed the same factor structure for both nurses and physicians. The *r* values of the test–retest reliability correlations were all 0·7 or above. Internal consistency was demonstrated by a Cronbach’s α = 0·8 or above.

**Conclusion:**

The Nurse–Physician Collaboration Scale can be used to establish standards for nurse–physician collaboration, to measure the frequency of collaborative activity, and to verify unit-specific relationships between collaboration and quality of care.

**What is already known about this topic**

- Previous research has focused on interactions and relationships between nurses and physicians, but there have been few measurements of specific behaviours associated with relationships in patient-centred care.
- Construct validity has been explored by an exploratory factor analysis of Nurse–Physician Collaboration Scales in earlier studies.
- There have been few attempts to verify a factor structure for a Nurse–Physician Collaboration Scale by simultaneous analysis of several groups.

**What this paper adds**

- The newly developed Nurse–Physician Collaboration Scale focuses on measurement of specific behaviours associated with relationships between nurses and physicians in actual patient-centred care situations.
- This Nurse–Physician Collaboration Scale has satisfactory reliability, demonstrated by Cronbach’s alpha coefficients (0·8 or above) and test–retest coefficients (0·7 pr above).
- As a result of simultaneous analysis of several groups and a confirmatory factor analysis, three dimensional factors were confirmed: sharing of patient information, joint participation in the cure/care decision-making process, and cooperativeness.

**Implications for practice and/or policy**

- The Nurse–Physician Collaboration Scale can be used for process evaluation by regularly measuring nurse–physician collaboration, and to identify relative differences in collaboration between medical institutions.
- Reviewing the relationship between responses to the Nurse–Physician Collaboration Scale and the quality of care will allow staff to recognize the importance of nurse–physician collaboration.
- This scale will also be effective for analysing factors that promote or hinder nurse–physician collaboration with regard to patient-centred care.

## Introduction

Today’s healthcare systems have become so complex that a division of labour among specialists in various fields has become indispensable. Interdisciplinary collaborative team care is required because only so much can be achieved by a single individual or group of professionals, as well as because the diverse needs of patients must be met 24 hours/day in a limited time (Kano 2000, Morita *et al.* 2005). The average length of hospital stay in Japan is currently being shortened in accordance with recent government guidelines, making collaboration more essential as critical decisions are compressed in time and patient turnover increases without increases in staff.

Since the entire nursing staff on patient units changes as often as monthly because of staff shortages, thinking about collaboration in terms of stable teams is inappropriate (Institute of Medicine 2003). The work of all staff members is governed by lines of authority and guided by institutional procedures. Although collaboration among healthcare professionals, here limited to nurses and physicians, is critical to patient care, it has been little studied in Japan.

Researchers in Europe and the United States of America (USA) have focused on collaboration among healthcare professionals and others, and have evaluated its impact on the quality of care and confirmed its importance. European and US healthcare institutions are trying to improve the quality of healthcare by strengthening such collaboration (Knaus *et al.* 1986, Shortell *et al.* 1994, Curley *et al.* 1998, Baggs *et al.* 1999, Gittell *et al.* 2000, Hinshaw 2002, Hamric & Blackhall 2007).

## Background

Research in Europe and the USA has focused on self-report measurements of collaboration and related concepts, mainly in relation to nurses and physicians. For example, the Collaborative Practice Scale (CPS) is based on the work of Blake and Mouton (1970), Thomas and Kilmann (1978) and Thomas (1982), theorists who focused on interaction methods using problem-solving or conflict management: assertiveness and cooperation. The Stichler Collaborative Behavior Scale (CBS) was developed using a conceptual framework relating to interactional theory and social theory (J.F. Stichler, University of Michigan, Ann Arbor, unpublished doctoral dissertation). Part 1 of the scale measures the amount of power balancing, interacting, and interpersonal valuing that occurs in a collaborative relationship. The Collaboration and Satisfaction About Care Decisions (CSACD) was developed by Baggs (1994), and its conceptual basis is the coordination theory of Thompson (1967) and Thomas (1976) for complex organizations, which expanded the collaboration attributes to four: shared responsibility for planning, shared decision-making, open communication and coordination.

The ICU Nurse–Physician Questionnaire (ICUN-P-Q) developed by Shortell *et al.* (1991) and the Relational Coordination developed by Gittell *et al.* (2000) are similar to instruments measuring cooperation. The ICUN-P-Q measures organizational climate, with a focus on unit culture, leadership, communication, coordination, problem-solving and conflict management. The concept of relational coordination was developed and validated in the context of commercial airline flight departures, and it is expected to be of value in achieving performance in settings that are highly uncertain, interdependent and time-constrained. The Relational Coordination Scale measures collaboration among physicians, nurses, physical therapists, and social workers, and encompasses four communication dimensions: frequency, timeliness, accuracy, and problem-solving, and three relationship dimensions: shared goals, shared knowledge and mutual respect.

Three of these scales, the CPS, CSACD and ICUN-P-Q, were developed to measure attitude toward cooperation among healthcare professionals, especially between nurses and physicians in clinical situations. In contrast, the CBS focuses on relationships between nurses and physicians by measuring the frequencies of cooperative actions. The Relational Coordination Scale emphasizes effectiveness of communication among healthcare professionals by asking detailed questions, but it does not measure specific behaviours associated with nurse–physician relationships in the process of patient-centred care.

The only measurements of nurse–physician collaboration in Japan have been from the viewpoint of nurses and obtained by means of the Nurses’ Perception of Physicians/Nurses Collaboration Scale (Ushiro & Nakayama 2005), which measures nurses’ self-assertiveness towards physicians, not nurse–physician collaboration. Its two dimensions are cooperativeness and self-assertiveness in relation to burnout and incidents. Other research in Japan (Fujino *et al.* 2004, Kataoka *et al.* 2005, Ishikawa *et al.* 2007) has measured the frequency of routine conferences to promote team cooperation in home-care and gynecological wards or psychiatric hospitals. However, the frequency of conferences is not always a sign of collaboration. The fact that conferences are held very frequently does not mean that the participants discuss matters freely and openly. Despite the fact that collaboration among healthcare staff is recognized as important, there have been no specific studies on the subject in relation to quality of care in Japan. Thus, while taking account of earlier research, the need to develop a scale to measure nurse–physician collaboration was confirmed.

Therefore a new scale, the Nurse–Physician Collaboration Scale (NPCS), was developed to allow study of the relationships between collaboration and quality of hospital care, to analyse factors that promote collaboration, and to devise collaborative system planning.

### Concepts of Nurse–Physician Collaboration Scale

The NPCS is based on the work of Simon (1977), Innami (2002), and Miyagawa (2004). All these theorists focused on information management processes that are used to solve problems or in decision-making, because information is closely linked to problem-solving and decision-making with regard to patient care. Simon’s idea, however, differs slightly from the ideas of the other theorists. Innami and Miyagawa suggested that there are three basic elements in the information management process: shared information, decision-making/consensus building and action. Since healthcare institutions are staffed by diverse professionals, it is especially important to solve patients’ problems from diverse standpoints. Thus, the concept of collaboration assumes the following three constructs: sharing of patient information, joint participation in the decision-making process, and cooperativeness.

#### Operational definition

For the purpose of the study, nurse–physician collaboration was defined as actions related to sharing information about patients, participating in decision-making concerning patient care, and providing comprehensive care to patients from a patient-centred perspective.

## The study

### Aim

The aim of the study was to develop and test the psychometric properties of the Nurse–Physician Collaboration Scale.

### Instrument development

The Nurse–Physician Collaboration Scale was devised by a step-by-step process that consisted of item design, item refinement, and testing for reliability and validity.

#### Item design

Items were designed on the basis of a sequential process that consisted of literature review; observation of nurse–physician exchanges in each unit/ward of three acute care hospitals in a large city in Japan; key-informant interviews of seven nurses and nine physicians from the same hospitals by means of a semi-structured format.

The interviews were designed to: (1) clarify whether physicians and nurses provide information (e.g. explanation) to patients and how physicians and nurses currently make decisions about cure/care, and clarify whether there are any problems with the decision-making process, and if so how this needs to be changed in the future and (2) determine the course of action in the decision-making process when opinions differed (e.g. between physician and nurse, between healthcare professional and patient).

Nine categories of items were created based on interviews and observations of physician–nurse interactions on the ward: (1) sharing of information concerning the patient’s condition, (2) mutual understanding of the patient’s feelings, (3) joint participation in planning, (4) common objectives, (5) joint resolution of problems, (6) trust and respect, (7) awareness of role and responsibility, (8) mutual support and (9) open communication. After reviewing some observation and interview records, the categories ‘sharing of information concerning the patient’s condition’ and ‘mutual understanding of patient’s feelings’ were combined into the category ‘sharing of patient information;’‘joint participation in planning,’‘common objective,’ and ‘joint resolution of problems’ were combined into the category ‘joint participation in the cure/care decision-making process;’ and ‘trust and respect,’‘awareness of role and responsibility,’‘mutual support,’ and ‘open communication’ were combined into the category ‘cooperativeness’.

Three constructs of Nurse–physician collaboration that were the basis for the item design were identified: sharing of patient information, joint participation in the cure/care decision-making process, and degree of cooperation; the resulting scale contained 69 items. Respondents were asked to rate each behaviour on a 5-point scale; (1) Always, (2) Usually, (3) Sometimes, (4) Rarely and (5) Never. The specific instructions were: ‘The purpose of this scale is to determine the extent of collaborative behaviours that generally exists between a single nurse/physician and other physicians/nurses with whom they work in providing patient care. For each statement circle (○) the box that indicates the frequency with which each behaviour occurs. Please answer each item as best you can’.

The goal was to design scale items that required respondents to imagine actual situations, thereby making it easy for them to respond. It was hoped that this procedure would result in fewer measurement errors.

#### Refinement

To refine the 69 items in the scale and ensure their validity, the content of each item was examined and pre-tested by taking two factors into account.

The first factor taken into account was the match rate between scale items and constructs; that is, whether each of the individual items and all of the items in general matched the constructs. Seven nurses (nursing management educators or nursing doctoral students) and four physicians (each with over 10 years of clinical experience) were asked to respond to the questionnaire. A scale item was rejected if the match rate between the construct and the item was less than 50%, when over half of those responded judged that the item did not correspond to the construct, or if anyone pointed out a problem in the wording of the item. Based on the responses by the physicians, six of the 32 items in the ‘sharing of patient information’ category had a match rate below 50%, no items in the ‘joint participation in the cure/care decision-making process’ category had a match rate below 50%, and three of the 16 items in the ‘cooperativeness’ category had a match rate below 50%. The results for whether each item and all items in general matched each of the three constructs showed that 50% of the physicians responded that there was either a ‘fair degree’ or a ‘high degree’ of correspondence for ‘sharing of patient information’ category, and 80% of the physicians responded that there was either a ‘fair degree’ or a ‘high degree’ of correspondence for the ‘joint participation in the cure/care decision-making process’ and ‘cooperativeness’ categories. However, two physicians pointed out that the wording of some of the items was ambiguous, that there were too many items, that some items were redundant, and that it was difficult to respond to a negative sentence. In addition, the following items were proposed by two physicians: (1) sharing of information on patient’s condition and treatment policy, (2) greetings between members of the staff with different occupations and (3) matters related to medical care accidents. Based on responses by the nurses, 13 of the 32 items in the ‘sharing of patient information’ category had a match rate below 50%; one of the 22 items in the ‘joint participation in the cure/care decision-making process’ category had a match rate below 50%; and one of the 15 items in the ‘cooperativeness’ category had a match rate below 50%. The results for whether each item and all items in general matched each of the three constructs showed that over 80% of the nurses responded that there was either a ‘fair degree’ or a ‘high degree’ of correspondence for all constructs. However, three nurses pointed out that the wording of some of the items was ambiguous, that there were too many items, that some items were redundant, and that it was difficult to respond to a negative sentence. In addition, the following items were proposed by three nurses: (1) decision-making in regard to the patient’s diet and repose, (2) taking each other’s schedule into account, and (3) matters related to medical care accidents.

The second point taken into account was the time required to respond to the questionnaire. To verify the quality of the questionnaire, eight nurses (with over 2 years of experience) and five physicians (with over 3 years of clinical experience) said that negative questions were hard to answer and that the number of items were too high to respond to in a short time. After the assessment, 64 nurses (average age, 28·9 ± 5·43 years) and 24 physicians (average age, 34·5 ± 6·55 years) made final refinements to the scale items by revising the content and wording based on the responses made by the physicians and nurses. For example, when the number of responses for a certain item was much higher than that for other items, the wording of the item was changed so that the distribution became closer to a normal distribution. Through this process, 51 items common to both nurses and physicians were obtained.

### Participants

Forty of the 87 acute care hospitals in a large city in Japan were randomly selected in January 2006, and the managers of 27 of these consented to their staff being asked to participate in the study. Questionnaires were mailed to 1584 nurses with two or more years of clinical experience and 843 physicians with three or more years of clinical experience at the 27 hospitals. Simple random sampling was performed using SPSS software, and data for testing came from samples of 27 of all 87 acute care hospitals listed by the Bureau of Social Welfare and Public Health, Metropolitan Government, Japan.

### Ethical considerations

The study was approved by the appropriate ethics review board. A letter of invitation outlining the aims and giving further details about the study accompanied each questionnaire. The questionnaires were sent to the heads or persons in charge in the hospitals or wards and distributed to each of their members. Consent to participate was assumed on the basis of a returned questionnaire, and the material returned did not contain any personal information that could be used to identify the respondent.

Test–retests were performed in the same manner as described above, with no participant names stated on the returned questionnaires. Only those participants who consented have written four random letters of the English alphabet on the upper right corner of the questionnaire, and the answered questionnaires were sent back by mail.

### Data analysis

Cronbach’s α coefficients and test–retest reliability coefficients were calculated to evaluate the internal consistency and stability of the scales. Alpha coefficients were also calculated for item-total (I-T) correlation and for item elimination. Construct validity was first confirmed by exploratory factor analysis, and then by confirmatory factor analysis. The confirmatory factor analysis was performed to confirm the degree of model-fit in both nurses’ and physicians’ factor models after the exploratory factor analysis. Finally, simultaneous analysis of several groups was performed to confirm factorial invariance, the same factor structure for both nurses and physicians. The following three models were compared: a single-factor model, a three-factor model, and a second-order three-factor model. Since the fit index values for the three-factor model (using the three constructs as subscales) and the second-order three-factor model (using the three constructs as one aggregate scale) were the same, the second-order three factor model was omitted.

Next, a model of error covariance, which corrects goodness of fit, was calculated (Kano 2002). To confirm the same factor structure for both nurses and physicians, simultaneous analysis of several groups was performed to assess factorial invariance. Finally, the Comparative Fit Index (CFI), the Root Mean Square Error of Approximation (RMSEA), and Akaike’s Information Criterion (AIC), a Japanease statistic (Toyoda 2007), were used to verify model fitness.

Convergent validity indicates that two measures that are thought to reflect the same underlying phenomenon will yield similar results or will correlate highly. Convergent validity of the NPCS was assessed by means of the Team Characteristic Scale developed by The Japan Institute of Labour (2003), a 22-item organization instrument used to verify whether team members share knowledge and information. A high value indicates that a team is functioning well. To verify concurrent validity it was necessary to calculate the negative correlation for cooperation and conflict between physicians and nurses, and the Intergroup Conflict Scale (Kawakami & Fujigaki 1996) was used to do this. This scale is part of the Japanese version of the Generic Job Stress Questionnaire published by the National Institute for Occupational Safety and Health (NIOSH) in Japan. Statistical analyses were conducted by using SPSS 16·0J and Amos 16.0 software (SPSS Japan Inc., Tokyo, Japan).

## Results

Questionnaires were returned by 1246 nurses and 459 physicians (response rate 78·7% for nurses and 54·4% for physicians). Valid responses were obtained from 1217 nurses (average age, 29·34 ± 6·05 years) and 446 physicians (average age, 37·07 ± 8·13 years).

### Correlations between items and no response items

Using the selection criteria proposed by Kano (2002), coefficients for the correlations between items were calculated. When the correlation coefficient between two items was 0·8 or above, a high correlation rate, one of them was deleted. When the coefficient was 0·7 or above, which falls within the cutoff range, indicating acceptability, deletion or retention of the item was considered. In addition, items to which no response had been made were considered for deletion.

### Exploratory factor analysis

Exploratory factor analysis was carried out using a principal factor method with promax rotation. This yielded three factors: sharing of patient information, joint participation in the cure/care decision-making process, and cooperativeness. Five items, however, were not shared in the responses of nurses and physicians, and there was one item with low communality. All six items were deleted to make the scales comparable, and the exploratory factor analysis was carried out again. The resulting 45-item scale was analysed by exploratory factor analysis (principal factor method, promax method), and 12 items were dropped because of low factor loading (0·4 below) or because they did not belong to any factors. As a result, 33 items and three factors were common to both physicians and nurses. Five items, however, were not shared between responses of nurses and physicians, and there was one item with low communality. All six were deleted to make the scales comparable, and the exploratory factor analysis was carried out again. As a result, ‘shared patient’s information’ consisted of nine items, ‘joint participation in the cure/care decision-making process’ consisted of 12 items, and ‘cooperativeness’ consisted of six items (see Table 1). In addition the items related to ‘trust and respect’ and ‘awareness of role and responsibility’ were deleted from the cooperativeness category.

**Table 1:** Nurse–Physician Collaboration Scale items, factors, and descriptive statistics

	Nurses			Physicians		
Factors and Items	*n*	Mean ± sd	Factor loading	*n*	Mean ± sd	Factor loading
		
Joint participation in the cure/care decision-making process	α = 0·923			α = 0·926		
(J12) The nurses and the physicians exchange opinions to resolve problems related to patient cure/care	1207	3·17 ± 1·0	0·881	436	3·52 ± 0·91	0·811
(J11) In the event of a disagreement about the future direction of a patient’s care, the nurses and the physicians hold discussions to resolve differences of opinion	1209	3·07 ± 1·08	0·864	435	3·60 ± 0·98	0·811
(J16) The nurses and the physicians discuss whether to continue a certain treatment when that treatment is not having the expected effect	1208	3·01 ± 1·12	0·764	440	3·02 ± 1·10	0·737
(J10) When a patient is to be discharged from the hospital, the nurses and the physicians discuss where the patient will continue to be treated and the lifestyle regimen the patient needs to follow	1202	3·31 ± 0·98	0·737	437	3·43 ± 0·97	0·696
(J13) When confronted by a difficult patient, the nurses and the physicians discuss how to handle the situation	1210	3·40 ± 1·05	0·713	438	3·86 ± 0·90	0·700
(J8) The nurses and the physicians discuss the problems a patient has	1209	2·91 ± 1·0	0·705	438	3·31 ± 0·95	0·750
(J6) The nurses and the physicians together consider their proposals about the future direction of patient care	1211	3·17 ± 1·05	0·673	439	3·37 ± 1·0	0·571
(J15) In the event a patient develops unexpected side effects or complications, the nurses and the physicians discuss countermeasures	1209	3·67 ± 1·02	0·580	440	3·83 ± 0·98	0·676
(J14) In the event a patient no longer trusts a staff member, the nurses and the physicians try to respond to the patient in a consistent manner to resolve the situation	1212	3·81 ± 0·94	0·498	438	3·96 ± 0·88	0·665
(C2) The future direction of a patient’s care is based on a mutual exchange of opinions between the nurses and the physicians	1204	3·18 ± 0·93	0·498	437	3·52 ± 0·85	0·632
(J3) The nurses and the physicians seek agreement on signs that a patient can be discharged	1204	3·59 ± 0·93	0·473	439	3·74 ± 0·91	0·431
(J18) The nurses and the physicians discuss how to prevent medical care accidents	1212	2·71 ± 1·18	0·463	440	3·48 ± 1·08	0·462
Sharing of patient information	α = 0·905			α = 0·911		
(S4) The nurses and the physicians all know what has been explained to a patient about his/her condition or treatment	1210	3·54 ± 0·92	0·794	440	3·58 ± 0·99	0·679
(S9) The nurses and the physicians share information to verify the effects of treatment	1212	3·50 ± 0·88	0·778	439	3·65 ± 0·88	0·801
(S7) The nurses and the physicians have the same understanding of the future direction of the patient’s care	1214	3·39 ± 0·96	0·702	439	3·65 ± 0·90	0·845
(S2) The nurses and the physicians identify the key person in a patient’s life	1215	3·58 ± 0·99	0·695	439	3·86 ± 0·97	0·707
(S8) In the event of a change in treatment plan, the nurses and the physicians have a mutual understanding of the reasons for the change	1217	3·62 ± 0·89	0·688	438	3·85 ± 0·85	0·793
(S10) The nurses and the physicians check with each other concerning whether a patient has any signs of side effects or complications	1213	3·63 ± 0·94	0·676	440	3·75 ± 0·93	0·563
(S6) The nurses and the physicians share information about a patient’s reaction to explanations of his/her disease status and treatment methods	1206	3·10 ± 0·98	0·656	437	3·25 ± 0·99	0·678
(S1) The nurses, the physicians, and the patient have the same understanding of the patient’s wish for cure and care	1212	3·46 ± 0·84	0·634	439	3·79 ± 0·82	0·550
(S11) The nurses and the physicians share information about a patient’s level of independence in regard to activities of daily living	1212	3·37 ± 0·93	0·583	440	3·59 ± 0·92	0·605
Cooperativeness	α = 0·800			α = 0·842		

(C12) The nurses and the physicians can easily talk about topics other than topic related to work	1203	2·84 ± 1·20	0·770	438	3·69 ± 1·09	0·879
(C11) The nurses and the physicians can freely exchange information or opinions about matters related to work	1202	3·15 ± 1·05	0·761	437	3·95 ± 0·91	0·796
(C7) The nurses and the physicians show concern for each other when they are very tired	1202	2·81 ± 1·14	0·607	437	3·06 ± 1·08	0·551
(C9) The nurses and the physicians help each other	1203	3·19 ± 0·97	0·602	436	3·79 ± 0·92	0·640
(C10) The nurses and the physicians greet each other every day	1205	4·24 ± 0·87	0·499	437	4·38 ± 0·75	0·649
(C8) The nurses and the physicians take into account each other’s schedule when making plans to treat a patient together	1203	3·41 ± 1·16	0·433	434	3·50 ± 1·0	0·447

J, joint participation in the cure/care decision making process; S, sharing of patient information; C, cooperativeness.

The correlations among the three factors were 0·692, 0·568 and 0·512 for nurses and 0·739, 0·572 and 0·473 for physicians.

### Reliability

#### Internal consistency and item-total correlation analysis

Cronbach’s α coefficients for the nurses’ responses to the NPCS were 0·905 for sharing of patient information, 0·923 for joint participation in the cure/care decision-making process, and 0·800 for cooperativeness. When Cronbach’s α coefficients of the item-total correlations were compared with those obtained when an item had been eliminated, no item was found to lower the coefficient value. The item-total correlation values were high, ranging from 0·423 to 0·787.

Cronbach’s α coefficients for the physicians’ responses to the NPCS were 0·911 for shared patient information, 0·926 for joint participation in the cure/care decision-making process and 0·842 for cooperativeness. When Cronbach’s α coefficients of the item-total correlations were compared with those obtained when an item had been eliminated, no items was found to lower the coefficient value. The item-total correlation values were high, ranging from 0·502 to 0·801.

#### Stability

The test–retest method was used to assess stability. The participants were 90 of the 105 nurses and 48 of the 56 physicians who gave their consent to undergo re-testing after initial completion of the NPCS. The interval between the first and the second test was 2–3 weeks. The test–retest correlation coefficients for nurses were 0·710 (*P*< 0·01) for sharing if patient information, 0·658 (*P*< 0·01) for joint participation in the cure/care decision-making process, and 0·676 (*P*< 0·01) for cooperativeness. The test–retest correlation coefficients for physicians were 0·624 (*P*< 0·01) for sharing patient information, 0·798 (*P*< 0·01) for joint participation in the cure/care decision-making process and 0·774 (*P*< 0·01) for cooperativeness.

### Validity

#### Construct validity

**Figure 1:**
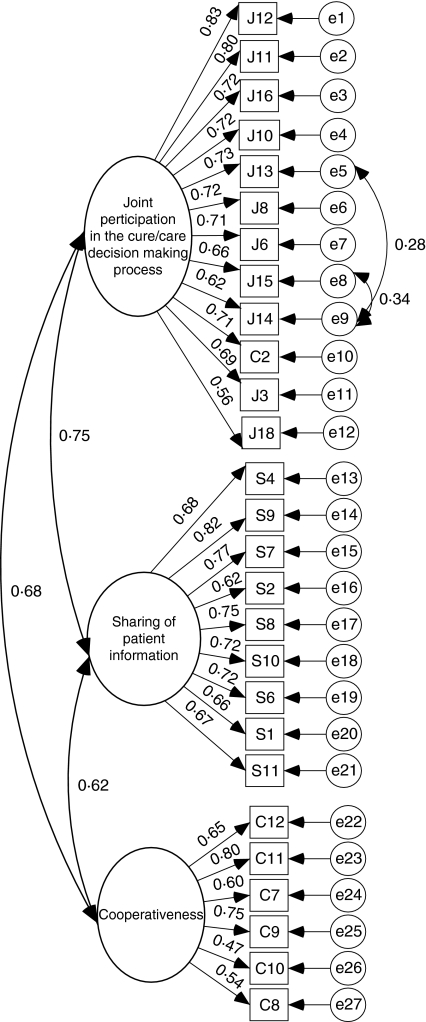
Confirmatory factor analysis: nurses (Error covariance correction model).

Exploratory factor analysis revealed that the NPCS has three dimensions. The scale was then assessed by CFA, which showed that both models had low goodness of fit values: CFI <0·8 and RMSEA >0·08 for the single-factor model, and CFI <0·9 and RMSEA <0·08 for the three-factor model. We therefore added the error covariance to the three-factor model by using the modification indices in Amos version 7·0 (see Figures 1 and 2), and the goodness of fit improved to CFI >0·9 and RMSEA <0·08 as a result. The error covariance correction model was therefore selected, and the factor loading values (path coefficients) obtained were statistically significant (*P*< 0·01).

**Figure:**
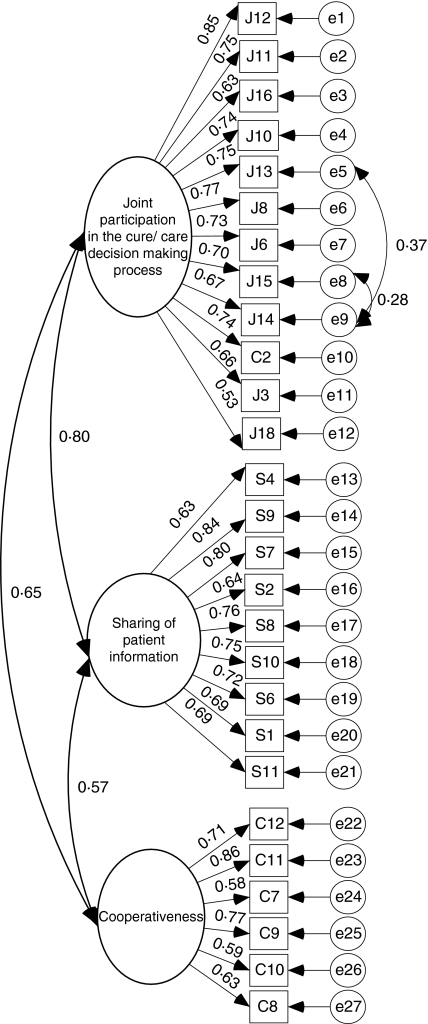
Confirmatory factor analysis: physicians (Error covariance correction model).

Simultaneous analysis of several groups was then performed on the error covariance correction model to identify the factor identity of the responses of nurses and physicians. Model 0 (configural invariance), model 1 (factor loadings equal), model 2 (factor loadings, factor variances, and covariance equal), and model 3 (factor loadings, covariance, and error variances equal) were used as the models for comparison. As shown in Table 2, model 2 with equality constraints for factor loading, variance, and covariance (shown by RMSEA = 0·046, AIC = 3115·888), yielded values smaller than the values for model 0, and thus was the correct result.

**Table 2:** Model Fit Statistics* for the Nurse–Physician Collaboration Scale (Error covariance correction model)

Model	CFI	RMSEA	AIC
Model 0†	0·905	0·047	3144·636
Model 1‡	0·906	0·046	3117·941
Model 2§	0·905	0·046	3115·888
Model 3¶	0·902	0·047	3203·008

CFI, Comparative Fit Index; RMSEA, Root Mean Square Error of Approximation; AIC, Akaike’s Information Criterion.

* Analysis: simultaneous analysis of several groups.

† Configural invariance.

‡ Factor loadings are equal.

§ Factor loadings, factor variances and covariances are equal.

¶ Factor loadings, covariance and error variances are equal.

#### Convergent validity

There were statistically significant positive correlations between the results obtained with the Team Characteristic Scale and with both the nurses’ responses (*r* = 0·360–0·523, *P*< 0·01) and physicians’ responses (*r* = 0·435–0·639, *P*< 0·01) to the NPCS.

#### Concurrent validity

Among the relationships between nurses’ responses to the NPCS and the Intergroup Conflict Scale, there were statistically significant negative correlations for all three factors (*r* = −0·20 to −0·236, *P*< 0·01). Among the relationship between physicians’ responses to the NPCS and the Intergroup Conflict Scale, there were statistically significant small negative correlations for shared patient’s information, (*r* = −0·165, *P*< 0·01) and cooperativeness. (*r* = −0·152, *P*< 0·01).

## Discussion

### Study limitations

The nurses and physicians who participated in this study were from hospitals located in a large city in Japan and the results might be different in other areas of Japan. The physician response rate was 54·4%, which means that opinions were received from only about half of the target group. Selection bias may be present because physicians who have a particular interest in Nurse–physician cooperation are more likely to have responded to the questionnaire. It should be pointed out that the physician response rate in similar studies in Japan is usually 20–30%, and thus the relatively high response rate in this study is a valuable asset.

While this study focused on collaboration between nurses and physicians with regard to patient care, it is also important to take into account the level of care and collaboration among the many other staff members involved. In one sense, then, measuring Nurse–physician collaboration has its limits. Although each hospital and ward has its own characteristics, collaboration in any hospital or ward is based on the role of each member of staff and their working habits. Assessing the medical professionals' content of collaboration provides a view of collaboration between nurses and physicians involved with patient care.

### Factor structure

Most measurement scales for ‘cooperation’ have been developed in Europe and the USA (Weiss & Davis 1985, J.F. Stichler, University of Michigan, Ann Arbor, unpublished doctoral dissertation, Heinemann *et al.* 1999,Hojat *et al.* 1999, Copnell *et al.* 2004), and the scales have focused on interaction and relationships. In other words, by emphasizing individual human relations, these scales measure the natural features or culture of an organization. The measurement scale developed in the present study, on the other hand, includes discussion and problem-solving elements between nurses and physicians, as in the CSACD and ICU N-P-Q.

However, ‘trust and respect’ and ‘awareness of role and responsibility’ were deleted from the cooperativeness category during the process of item selection because it was impossible to distinguish these items from those included in the ‘joint participation in the cure/care decision-making’ category. In other words, without a certain degree of mutual respect for each other’s field of expertise and mutual awareness of each other’s role, there can be no ‘joint participation in the cure/care decision-making process’. Thus, a better and more useful measure of collaboration would be to include items that describe specific Nurse–physician actions in the cooperativeness category instead of items such as ‘trust and respect’ and ‘awareness of role and responsibility’.

Three factors were derived for the NPCS as a result of the exploratory factor analysis and confirmatory factor analysis: sharing of patient information, joint participation in the cure/care decision-making process, and cooperativeness. Simultaneous analysis of several groups confirmed the factorial invariance (*a*(*b*) = *a*(*g*), ϕ(*b*) = ϕ(*g*)) of the NPCS for both nurses and physicians. These results showed that both nurses and physicians understand that they collaborate in the wards by sharing patient information, participating jointly in the cure/care decision-making process, and cooperating.

### Reliability and validity

The α coefficients of 0·8 and above indicate that these scales are internally consistent. All results for test–retest reliability were satisfactory, except for the physician responses regarding sharing of patient information (0·629). However, other α values were 0·7 or more, which confirms the stability of the scales. The results of the analysis strongly suggest that the NPCS is reliable and valid.

Based on the confirmation of factorial invariance and the positive correlation between the results obtained using our scale and the Team Characteristic Scale, it can be concluded that convergent validity was supported. Similarly, the negative correlation with the Intergroup Conflict Scale in the assessment of concurrent validity indicates that Nurse–physician conflicts can be controlled to some degree.

## Conclusion

In the future, it will be necessary to broaden the scale of research to hospitals in other cities in order to determine whether the same factors will be extracted and whether their reliability and validity can be verified. It will then be necessary to examine the relationship between collaboration and the quality of patient care by means of a hospital-by-hospital analysis based on nurse–physician relations in specific units/wards. Factors that aid or hinder collaboration should be identified. Finally, the items in the instrument may provide guidance for promoting collaborative relationships between nurses and physicians and staff in the many other occupations involved in patient care.
